# A potential decision-making algorithm based on endoscopic ultrasound for staging early gastric cancer: a retrospective study

**DOI:** 10.1186/s12885-022-09870-0

**Published:** 2022-07-13

**Authors:** Yan Yan, Zhonghua Ma, Xin Ji, Jiawei Liu, Ke Ji, Shijie Li, Qi Wu

**Affiliations:** 1 Key Laboratory of Carcinogenesis and Translational Research (Ministry of Education), Department of Endoscopy, Peking University Cancer Hospital & Institute, #52 Fucheng Road Haidian District, Beijing, China; 2 Key Laboratory of Carcinogenesis and Translational Research (Ministry of Education), Center of Gastrointestinal Cancer, Peking University Cancer Hospital & Institute, Beijing, China

**Keywords:** Endoscopic ultrasound, Gastric cancer, T staging

## Abstract

**Background:**

Clinical staging of gastric cancer (GC) before treatment is essential. Endoscopic ultrasound (EUS) is a recommended staging tool, but its efficacy remains controversial. Our previous prospective study evaluated the potential value of EUS for T staging and presented discrepancies. In this study, we aimed to evaluate the efficacy of EUS in T staging by comparing it with pathological staging. We analyze the factors that can potentially affect accuracy to identify suitable subgroups for EUS staging.

**Methods:**

Data from a total of 1763 consecutive patients with GC from January 2015 to December 2017 were analyzed. Results from EUS and pathological T staging were compared. The factors that might affect EUS’s accuracy were analyzed.

**Results:**

The sensitivity, specificity, positive predictive value, and negative predictive value of EUS in patients with early GC were 62.08%, 96.13%, 90.94%, and 80.21%, respectively. The accuracy rates of uT1, uT2–uT4, and uT3–uT4 were 90.94%, 79.02%, and 78.39%, respectively. In multivariate analysis, underestimation was more likely to be observed in patients with tumors located in the middle or upper third of the stomach. Overestimation was more likely to be observed in patients with tumors located in the lower third or those without ulcer. Other factors affecting accuracy included ulcer, differentiation, larger size and undergoing surgery.

**Conclusion:**

Our findings highlight the role of EUS in determining the T staging of GC. Overestimation and underestimation in T-staging were significantly associated with the tumor location in early GC, and a decision-making algorithm was proposed for clinical practice in early cancers based on these findings.

## Introduction

Accurate preoperative staging of gastric cancer (GC) is crucial for formulating precise therapeutic strategies [1, 2]. Emerging methods have been used for this purpose [2–4]. Endoscopic ultrasound (EUS) was first introduced in clinical practice in the 1980s. Current evidence has identified the efficacy of EUS as an important diagnostic modality for evaluating lesions of the digestive tract, with a high accuracy rate of approximately 90% [5, 6]. These findings indicate the role of EUS in assessing pretreatment T staging. However, the results reported by other researchers are considerably different [7, 8]. Of note, studies regarding the role of EUS in determining T1a, T1b, and advanced GC (AGC) are still limited, especially AGC. Therefore, there is an urgent need to determine the accuracy of EUS in T staging and to attach more importance to both early GC (EGC) and AGC.

Our previous study first implicated in vitro studies to determine the accuracy of EUS for early and advanced gastric carcinomas [6]. EUS was performed on gastric carcinoma specimens from 60 consecutive patients. It was found that the tumors located in the upper third of the stomach were expected to be diagnosed more accurately. Meanwhile, the accuracy of EUS displayed no significant correlation with histology, Lauren classification, and tumor location. Generally, the implication of standardized EUS scanning and high-quality images can lead to improved EUS accuracy [6], just like some previous reports [9]. However, the sample size used in our previous study was limited. No statistical differences were observed, which may be attributed to the limited sample size. In addition, the study setting was ideal; thus, the clinical value of EUS staging can hardly be affirmed by the results. We found some special cases in previous studies that cannot be properly staged because of tumor invasion patterns; thus, we aim to conduct more studies.

Here, we aimed to evaluate the efficacy of EUS in determining T stage by comparing it with pathological staging and to analyze the factors potentially correlated with EUS staging accuracy. In this study, we attempted to identify suitable subgroups for EUS staging.

## Materials and methods

### Patients

The data were collected from patients who were diagnosed with histology-confirmed gastric adenocarcinoma as the only type of primary cancer and underwent EUS between January 2015 and December 2017 at Peking University Cancer Hospital and Institute. Patients with a history of neoadjuvant therapy or previous endoscopic resection or surgery were excluded. The final pathological diagnosis was based on a completely resected specimen, and other malignancies, such as lymphoma and neuroendocrine tumor, were excluded. Histopathological evaluation was conducted based on the 8th edition of the American Joint Committee on Cancer tumor-node-metastasis cancer staging system.

All patients or their families provided written informed consent before undergoing any examination or treatment. This study was approved by the Beijing Cancer Hospital Research Ethics Committee and conducted according to the guidelines of the Declaration of Helsinki (2014KT11).

### EUS staging and histopathology

An echo-endoscope (GF-UE260-AL5, Olympus Corporation, Tokyo, Japan, or EG-530UR, Fujifilm Corporation, Tokyo, Japan) was used in this study. EUS was performed before treatment. The EUS procedures were performed by three qualified endoscopists. A qualified endoscopist is defined as an endoscopist with successful 225 EUS procedures in minimum [10]. The quality of the EUS images was assessed by endoscopists by reviewing the images and videos, in terms of proper placement of the probe and clarity of different layers. In EUS, the degree of tumor penetration into the gastric wall was categorized according to the deepest layer invasion. Detailed information regarding the definition of uT1a, uT1b, uT2, uT3, uT4a, and uT4b was described, as previously reported [6, 7]. In EUS imaging, the normal gastric wall would present as five layers which are marked as layer 1–5 corresponding with the mucosa, muscularis mucosae, submucosa, muscularis propria and serosa. Detailed tumor penetrating is identified as below: 1) uT1a, a hypoechoic expansion or thickening of layers 1 and 2 without interruption to the third layer; 2) uT1b, normal structures of the first to third was involved or destructed; 3) uT2, a dark expansion of layers 1‑4, which means tumor penetrates into the muscularis propria; 4) uT3, all layers cannot be distinguished, and the hypoechoic area has an relatively smooth border. These signs indicate invasion of the subserosa; 5) uT4a, all layers of the gastric wall are invaded, and the outer bright line is interrupted in an irregular pattern; this sign represents invasion of the serosa; and [6] uT4b, extension of the hypoechoic mass into surrounding organs such as the pancreas, liver or spleen.

The resected specimens were fixed in a formaldehyde solution for 12–24 h. Endoscopic specimens were serially sectioned at a 2–3-mm interval, while surgical specimens were routinely sectioned by pathologists.

### Variables

The collected variables included sex, age at diagnosis, primary tumor location, tumor size, degree of differentiation, Lauren classification, EUS T stage, pathologic T stage, and treatment method. For patients with EGC, gross type and ulceration status were also collected. Age was regrouped into ≤ 60 years and > 60 years. The location was grouped into the upper, middle, or lower third of the stomach according to the Japanese Gastric Cancer Association. Size was divided into ≤ 2.0 cm and > 2.0 cm groups. Well-differentiated or moderately differentiated gastric carcinoma was classified as differentiated type, whereas poorly differentiated gastric carcinoma was classified as undifferentiated type (including signet ring cell). The Lauren type was also recorded.

### Statistical analysis

Continuous data are presented as means and standard deviations, whereas categorical data are presented as numbers and proportions (%). Pearson’s χ^2^ test was used to analyze categorical variables. Multivariable analyses for potential factors affecting the outcomes (concordance rate) were performed using multiple logistic regression model. No covariates included in the regression models had missing values. The concordance rate and 95% CIs were calculated. Comparisons were made between the results of EUS T staging and pathological T staging for both early and advanced cases, separately. In the early stage, more variables were compared according to the criteria of endoscopic resection. We compared characteristics of pT1a and pT1b cases separately in uT1a/uT1b subgroup, as well as characteristics of uT1a and uT1b cases in pT1a cases. Statistical analysis was performed using Stata statistical software version 14.0 (StataCorp LP, College Station, TX, USA). The tests for significance were two-tailed, and a P value < 0.05 was considered statistically significant.

## Results

### Patient baseline characteristics

A total of 1763 patients underwent EUS staging and resection at our center between January 2015 and December 2017. Among them, 1302 patients with complete clinical and pathological data were included and analyzed in this study. The average age of the patients was 58.7 years. Male patients accounted for 70.4% of the 1302 patients. Ulcers accounted for 50.3% of the entire group. Moreover, the numbers of patients with intestinal, diffuse, and mixed types were 557, 353, and 392, respectively. Further, 31.2% of these samples were defined as differentiated type, and the rest were classified as undifferentiated. The detailed baseline characteristics of the patients are outlined in Table 1.

### The T-stage distribution

As depicted in Tables 1 and 2, 501 and 801 patients had EGC and AGC, respectively. Based on pathological T staging, pT1a, pT1b, pT2, pT3, pT4a, and pT4b were observed in 262, 239, 183, 337, 270, and 11 patients, respectively. Meanwhile, based on T staging by EUS, uT1a, uT1b, uT2, uT3, uT4a, and uT4b were observed in 148, 194, 301, 314, 343, and 2 patients, respectively. The detailed records of the EUS and pathological T staging distributions are presented in Table 2.

### The accuracy of EUS

In this study, we aimed to determine the accuracy of EUS T staging (clinical staging before treatment). As shown in Table 3, the accuracy rates of T1, T2–T4, and T3–T4 were 90.94%, 79.02%, and 78.39%, respectively. Further, considering the clinical decision-making process, the critical information preferentially required to be known in clinical practice was to identify whether the patient had EGC or AGC. Thus, the patients were divided into the EGC and AGC groups to further detect the potential of EUS T staging. To assess the ability of EUS to screen out and clinical identify EGCs, we analysed sensitivity (the proportion of uT1 in pT1 cases), specificity (the proportion of uT2-4 in pT2-4 cases), positive predictive value (proportion of pathological EGC cases(pT1) in which EUS diagnosed as EGCs (uT1)) and negative predictive value (proportion of pathological AGC cases (pT2-4) in which EUS diagnosed as AGCs(uT2-4)). As shown in Table 3, the sensitivity, specificity, positive predictive value, and negative predictive value of EUS were 62.08%, 96.13%, 90.94%, and 80.21%, respectively (Table 3). In detail, 90.94% of patients who were staged as EGC using EUS were found to have EGC based on pathological staging. Similarly, 80.21% of patients who were staged as AGC through EUS were confirmed pathologically.

### Detailed staging for early cases

**Fig. 1:**
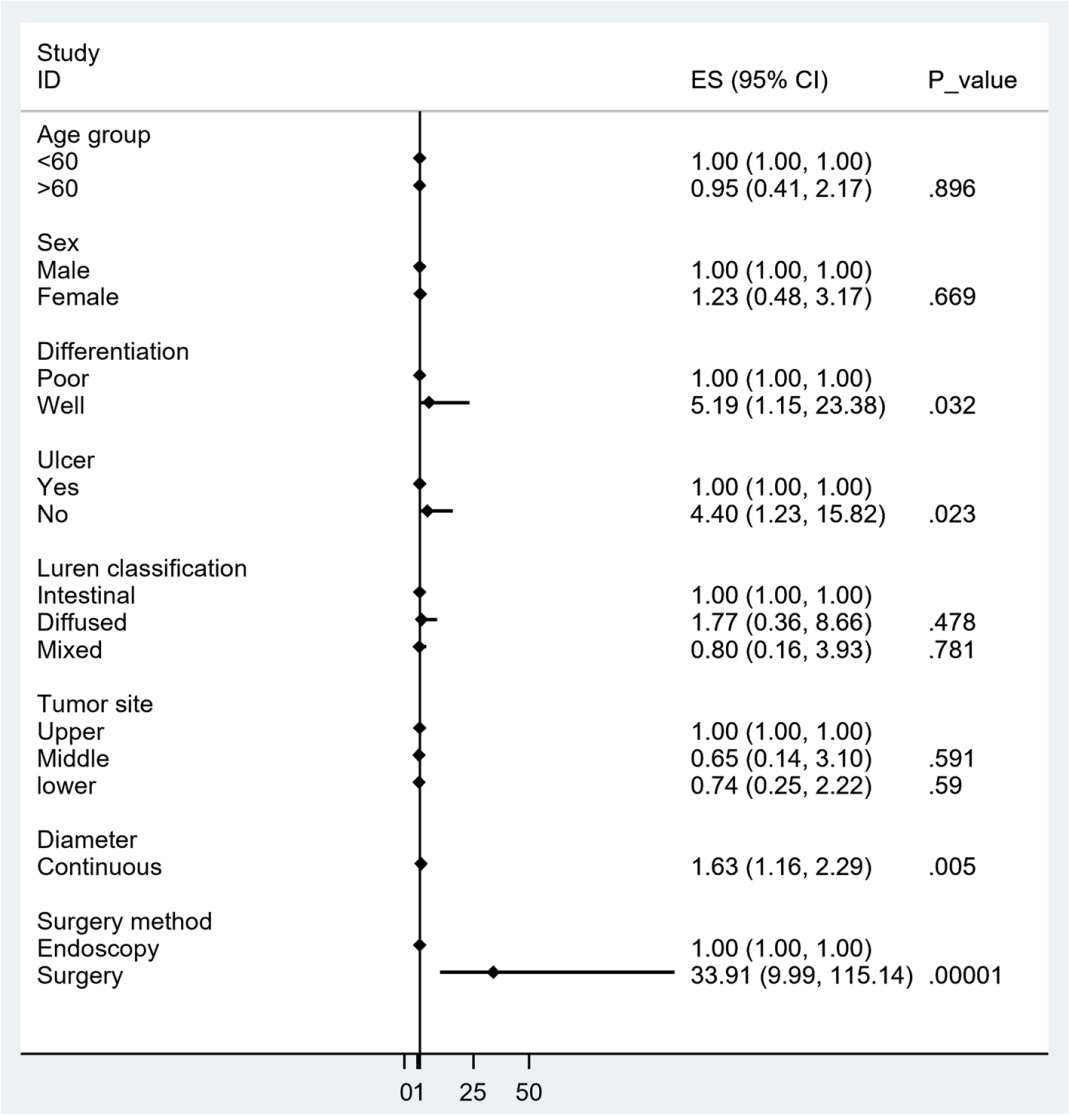
Forest graph of factors affecting accuracy in patients with pathological stage T1a

For patients with EGC, detailed T staging plays an important role in clinical decision-making. Therefore, we performed a multivariate logistic regression analysis to determine the factors affecting the accuracy of detailed T staging. As shown in Table 4, multivariate logistic regression analysis was performed in patients with pT1a, which can be divided into the uT1a and uT1b groups (Table 4 and Fig. 1). Compared patients with pT1a lesions diagnosed as uT1a, features of patients in the overestimated group (uT1b) were analyzed. Features such as differentiated histology, no ulcer, larger diameter or underwent surgery were of significant statistical difference and OR value > 1. In other words, patients who had differentiated histology, with no ulcer, with larger diameter tumors, or who underwent surgical treatment were more likely to be classified into the uT1b group, which means that EUS staging was overestimated.

**Fig. 2:**
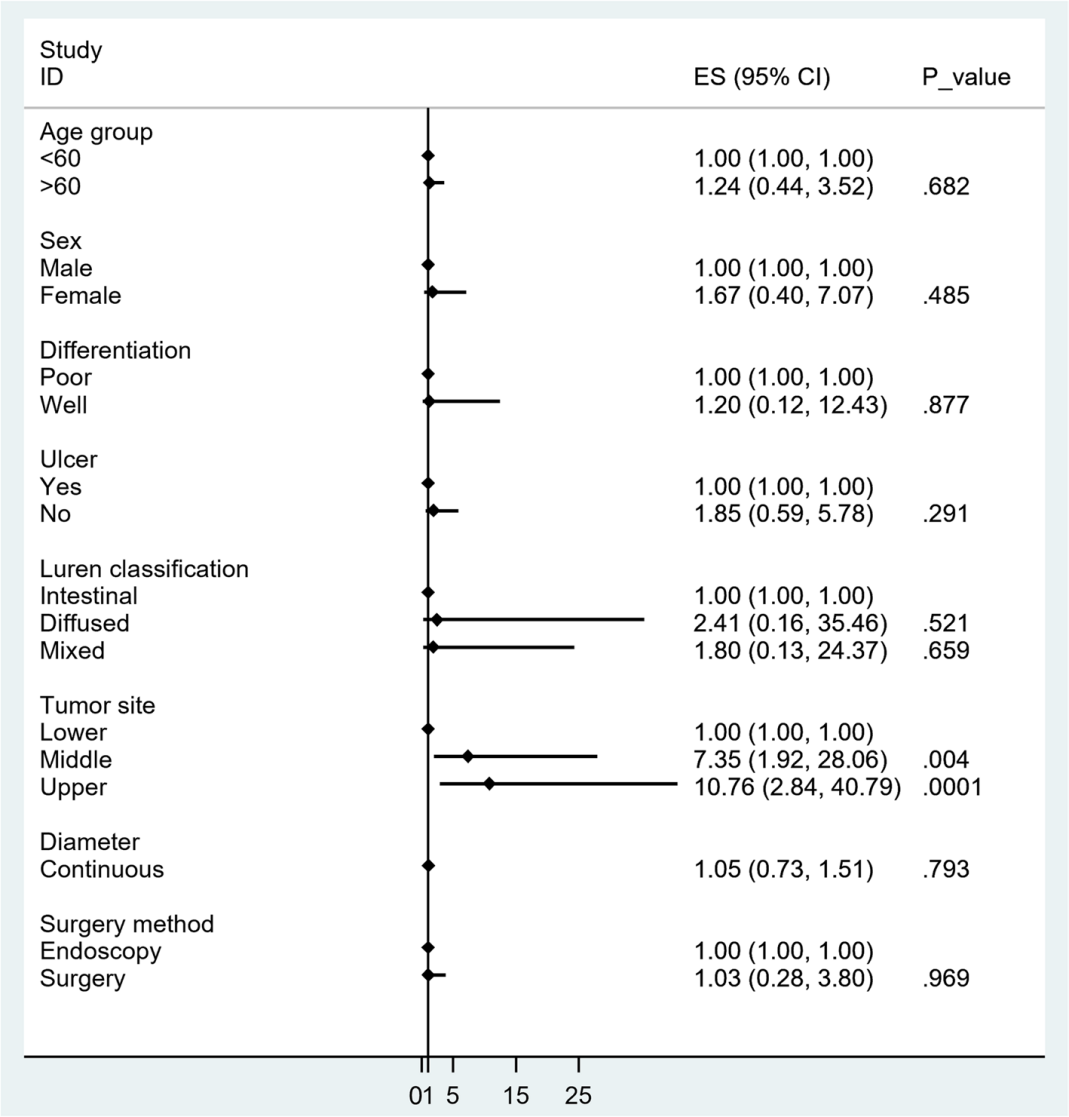
Forest graph of factors affecting accuracy in patients with ultrasonic stage T1a

**Fig. 3:**
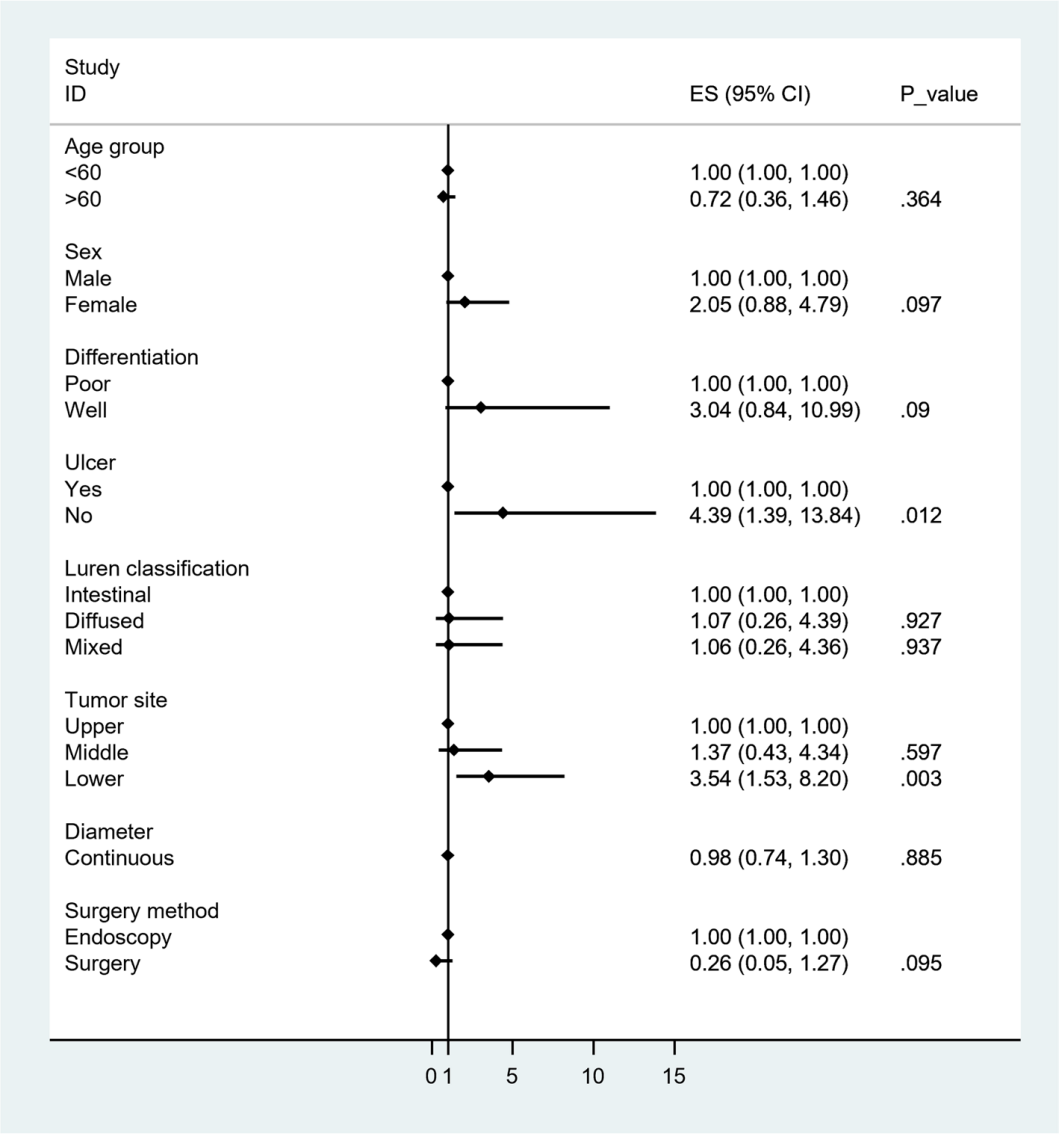
Forest graph of factors affecting accuracy in patients with ultrasonic stage T1b

Subsequently, we found that underestimation was more likely to be observed in patients with upper or middle GC than in those with lower GC. Therefore, it may be of great significance for patients with upper or middle GC and uT1a to have further concerns, thereby avoiding incomplete ESD(Endoscopic submucosal dissection) (Table 5 and Fig. 2). Next, multivariate logistic regression analysis was performed in patients with uT1b, which can be divided into the pT1a and pT1b groups. The results indicated that overestimation was more likely to be observed in patients with lower GC without ulcers (Table 6 and Fig. 3).

**Fig. 4:**
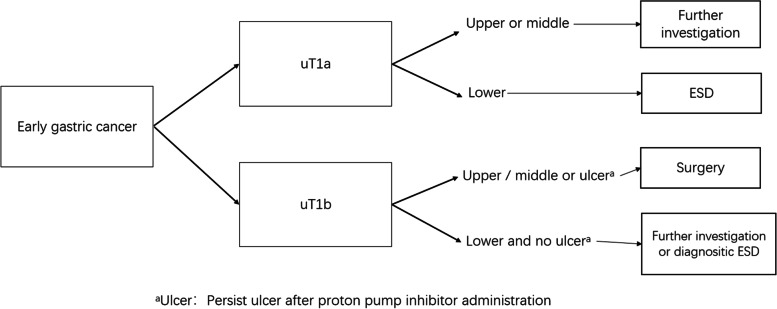
A potential decision-making algorithm based on EUS for early gastric cancer

As depicted in Tables 5 and 6, for EGC, with uT1a diagnosis, the tumors located in the upper or middle part of the stomach were more likely to be underestimated using EUS (Figs. 2 and 3). Endoscopic submucosal resection was recommended if patients with lower GC were diagnosed with uT1a using EUS. Specifically, further examination was strongly recommended for upper or middle GC if the result obtained from EUS was determined to be uT1a. Moreover, surgical operations were highlighted in patients with upper or middle GC and EUS diagnosis of uT1b. Regarding the lower tumor, ultrasonic T staging tended to be overestimated. Patients with lower GC diagnosed with uT1b through EUS were advised to undergo full assessments to obtain more accurate results. Further, endoscopic resection was considered if patients with a lower GC were diagnosed as having uT1b using EUS. The recommended process is highlighted in the following flowchart found in Fig. 4.

## Discussion

Emphasis on clinical staging has been increasing in recent years [11], and obtaining precise and reliable clinical staging is a challenging topic in the real world [12]. EUS was used as a standard procedure in preoperative T staging of patients with gastric carcinomas. For GC, as EUS can visualize the different layers of the gastric wall as corresponding sonographic layers, it is generally considered an effective tool for T staging [13, 14], especially in detailed staging——early (T1) vs advanced cancers; T1a vs T1b cancers [14–18]. However, in previous studies, the accuracy rate of EUS ranged from 43% to more than 90% [15, 19]. In addition, several meta-analyses have also identified remarkable heterogeneous results [5, 20]. Therefore, the validity of EUS for staging remains controversial.

Our previous prospective study confirmed the efficacy of EUS for T staging [6]. The deepest point determined by EUS showed great consistency with pathological findings. We tried to elucidate the answer for the heterogeneity. One reason might be related to the procedure. As mentioned in previous reports, the image quality and standardization no-omitting EUS scanning may be of great significance in increasing the accuracy of EUS [9, 16]. The experience of operator also affect the accuracy [21]. In our center, a great number of patients with GC undergo EUS before any treatment for staging. And quality of EUS procedure and image is routinely evaluated. The other one reason might be related to the tumor. We found some interesting cases with representative discrepancies and these inconsistencies were reported. Some tumors tend to infiltratively invade the layers without destruction, which could result in incorrect pre-T staging and inappropriate treatments.

In this study, we retrospectively analyzed the data of 1736 patients, of whom 1302 patients were evaluated as having resectable tumors and underwent resection (endoscopic or surgical) as primary treatment. A comparison was made between the EUS and pathological T stages. Determining a case as early or advanced was the first step in staging tumors. Our results suggested that the specificity and positive predictive value of EUS were 96.13% and 90.94%, respectively. These findings, together with the previous studies, highlight the value of EUS in determining T staging, especially in distinguishing between early and advanced GC. Subsequently, treatment was decided. T3 and T4a are difficult to distinguish, as the serosa is significantly thin to detect invasion. However, it does not have a significant effect on treatment choices. Generally, either cT3 or cT4a is appropriate for neoadjuvant therapy before surgery.

For EGC cases, the most important issue is the accurate identification of ESD candidates which is mainly based on T staging. Therefore, we aimed to distinguish between the “fit” subgroup for EUS staging and the “un-fit” subgroup for EUS staging in EGC cases. Therefore, we performed a multivariate logistic regression analysis between the “right” and “wrong” groups. When we examined pT1a cases (uT1a and uT1b in pT1a), potential factors reported in previous studies were analyzed. Of note, lesion diameter, tumor differentiation, and ulcer showed statistical significance.

Underestimation in larger size is not surprising [9, 22–26]. Interestingly, the treatment choice showed significant statistical significance in uT1a and uT1b in pT1a cases (*P* < 0.001). In our center, ESD specimens are evenly spread and sectioned continuously in pathological processes. However, the surgical specimen was not. The specimens could not be sectioned thoroughly. Whether the deepest point is “caught” remains uncertain, especially for large ones. This may have led to the discrepancy. To the best of our knowledge, in most clinical centers in China, the pathological process for surgical specimens is not as thorough as that for ESD specimens, because of the large size of the specimen and the shortage of pathologists. In advanced cases, pathologists find the deepest invasion part easier, but in early cases, this is not the case. Because the specimens are not spread evenly when fixed, identifying the deepest point by the naked eye seems relatively difficult.

When we examined uT1a and uT1b cases (pT1a and pT1b in uT1a, pT1a and pT1b in uT1b), ulcer status and location showed statistically significance. Ulcers might mimic tumors in some EUS images and lead to incorrect estimation, as previously reported [24, 27–29]. However, in our center, early cancers with ulcers are treated with proton pump inhibitor before staging to accelerate healing [30], possibly making up for this deficiency. After healing, the ulcer subgroup showed equally or better accuracy for pretreatment staging. It is also reported, even EUS’s accuracy was poor, it was still superior to that of conventional endoscopy in ulcerative EGC [29].

According to our results, tumor location matters. If the lesion is located in the antrum, uT1a is more likely to be pT1a, and ESD is recommended. uT1b might also be pT1a eventually, which we recommend further evaluation or diagnostic ESD. When a lesion in the upper stomach is found to be uT1b, surgery is recommended; otherwise, we could perform diagnostic ESD or further investigation. Similar findings are also mentioned by some other scholars in that lesions located in the upper stomach tend to be underestimated [9, 26, 31]. For further investigation, we routinely use hypotonic air-filling contrasted-enhanced computed tomography and enhanced endoscopic imaging such as magnification. From the data of our centre, hypotonic air-filling contrasted-enhanced CT in gastric window provides more accurate information for staging in early gastric cancer than conventional CT in abdominal window [32]. Based on these findings, we proposed a decision-making algorithm, as shown in Fig. 4. This finding is consistent with those of previous reports and our clinical impressions.

In recent years, patients with locally advanced cancers mostly receive neoadjuvant therapy before resection in China, so we cannot compare the pathological result with EUS in this new era. But treatment choice and response assessment are of unprecedented importance in this new era, thus we need a reliable tool for detailed staging. Also as mentioned in a previous study, EUS may provide a highly informative assessment of gastric wall invasion [12]. Therefore, we selected a series of consecutive patients during the period from 2015 to 2017. In our data, the proportion of advanced GC is remarkable. For patients with early cancers, we present a large proportion who underwent surgery. However, this study has limitations in that only a single center’s data were analyzed in this study and no mini-probe EUS was used. Additionally, this was a retrospective study, and prospective studies should be conducted in the future to validate or revise this potential algorithm.

## Conclusions

Our findings highlight the role of EUS in determining T staging of GC, including EGC and AGC. Overestimation and underestimation of T staging were significantly associated with the tumor location, ulcer or not, differentiation and size. Importantly, patients with EGC required to undergo surgical operations should be provided significant emphasis on pathological evaluation. For patients with EGC who candidates for ESD were, it may be convenient and helpful to use the simple algorithm based on tumor location and presence of persist ulcer after PPI treatment.

**Table 1:** Clinical characteristics of 1302 patients with gastric cancer

	N	%
Sex		
Male	916	70.4
Female	386	29.6
Age mean (SD) (years)	58.7 (10.7)	
Location		
Upper	375	28.8
Middle	249	19.1
Lower	672	51.6
linitis plastica	6	0.5
Size (cm)		
≤ 2.0 cm	400	30.7
> 2.0 cm	902	69.3
Gross type		
I	17	1.3
IIa	39	3.0
IIb	90	6.9
IIc	256	19,7
III	99	7.6
AGC^a^	801	61.5
Ulcer		
Yes	655	50.3
No	647	49.7
Lauren classification		
Intestinal	557	42.8
Diffused	353	27.1
Mixed	392	30.1
Histology		
Differentiated	406	31.2
Undifferentiated	896	68.8
Treatment		
ESD^b^	120	9.2
Surgery	1182	90.8

^a^ *AGC* advanced gastric cancer, ^b^ *ESD* endoscopic submucosal dissection

**Table 2:** Ultrasound and pathological T staging distribution of 1302 patients

EUS^a^ T stage	Pathological T stage						
	pT1a	pT1b	pT2	pT3	pT4a	pT4b	total
T1a	122	25	1	0	0	0	148
T1b	76	88	22	3	5	0	194
T2	43	92	84	55	27	0	301
T3	16	26	55	119	93	5	314
T4a	5	8	21	159	144	6	343
T4b	0	0	0	1	1	0	2
total	262	239	183	337	270	11	1302

^a^ EUS, endoscopic ultrasound

**Table 3:** The accuracy rates of different EUS stages in 1302 patients and sensitivity, specificity, positive and negative predictive values of EUS for early gastric cancer^a^

T stage by EUS	Accuracy rate, %	95% CI
uT1a	82.43	75.33–88.19
uT1b	45.36	38.22–52.65
uT1	90.94	87.38–93.76
uT2-T4	79.02	76.30–81.56
uT3-T4	78.39	75.04–81.48
Sensitivity^b^	62.08	57.67–66.34
Specificity ^c^	96.13	94.55–97.36
Positive predictive value^d^	90.94	87.38–93.76
Negative predictive value^e^	80.21	77.54–82.69

^a^Early gastric cancer means pathological T1,

^b^ Sensitivity is defined as uT1 in pT1

^c^ Specificity is defined as uT2-4 in pT2-4

^d^ Positive predictive value is defined as pT1 in uT1

^e^ Negative predictive value is defined pT2-4 in uT2-4

**Table 4:** Multivariate logistic regression analysis of accuracy in patients with pathological stage T1a

Variables	Multivariate OR^a^	95% CI	*p* value
Age			
< 60	1 (reference)		
> 60	0.95	0.41–2.17	0.896
Sex			
Male	1 (reference)		
Female	1.23	0.48–3.17	0.669
Histology			
Differentiated	1 (reference)		
Undifferentiated	5.19	1.15–23.38	0.032^*^
Ulcer			
Yes	1 (reference)		
No	4.40	1.23–15.82	0.023^*^
Lauren classification			
Intestinal	1 (reference)		
Diffused	1.77	0.36–8.66	0.478
Mixed	0.80	0.16–3.93	0.781
Tumor location			
Upper	1 (reference)		
Middle	0.65	0.14–3.10	0.591
Lower	0.74	0.25–2.22	0.590
Diameter	1.63	1.16–2.29	0.005^*^
Treatment			
ESD	1 (reference)		
Surgery	33.91	9.99–115.14	< 0.001^*^

^a^ OR value, > 1 means the group were more likely to be overestimated

**Table 5:** Multivariate logistic regression analysis of accuracy in patients with ultrasonic stage T1a

Variables	Multivariate OR	95% CI	*p* value
Age			
< 60	1 (reference)		
> 60	1.24	0.44–3.52	0.682
Sex			
Male	1 (reference)		
Female	1.67	0.40–7.07	0.485
Histology			
Differentiated	1 (reference)		
Undifferentiated	1.20	0.12–12.43	0.877
Ulcer			
Yes	1 (reference)		
No	1.85	0.59–5.87	0.291
Lauren classification			
Intestinal	1 (reference)		
Diffused	2.41	0.16–35.46	0.521
Mixed	1.80	0.13–24.37	0.659
Tumor location			
Lower	1 (reference)		
Middle	7.35	1.92–28.06	0.004^*^
Upper	10.76	2.84–40.79	< 0.001^*^
Diameter	1.05	0.73–1.51	0.793
Treatment			
ESD	1 (reference)		
Surgery	1.03	0.28–3.80	0.969

**Table 6:** Multivariate logistic regression analysis of accuracy in patients with ultrasonic stage T1b

Variables	Multivariate OR	95% CI	*p* value
Age			
< 60	1 (reference)		
> 60	0.72	0.36–1.46	0.364
Sex			
Male	1 (reference)		
Female	2.05	0.88–4.79	0.097
Histology			
Differentiated	1 (reference)		
Undifferentiated	3.04	0.84–10.99	0.090
Ulcer			
Yes	1 (reference)		
No	4.39	1.39–13.84	0.012^*^
Lauren classification			
Intestinal	1 (reference)		
Diffused	1.07	0.26–4.39	0.927
Mixed	1.06	0.26–4.36	0.937
Tumor location			
Upper	1 (reference)		
Middle	1.37	0.43–4.34	0.597
Lower	3.54	1.53–8.20	0.003^*^
Diameter	0.98	0.74–1.30	0.885
Treatment			
ESD	1 (reference)		
Surgery	0.26	0.05–1.27	0.095

## Data Availability

The data that support the findings of this study are available from Peking University Cancer Hospital & Institute but restrictions apply to the availability of these data, which were used under license for the current study, and so are not publicly available. Data are however available from the authors upon reasonable request and with permission of Peking University Cancer Hospital & Institute.

## Abbreviations

AGC: Advanced gastric cancer
EGC: Early gastric cancer
EUS: Endoscopic ultrasound
GC: Gastric cancer
ESD: Endoscopic submucosal dissection
